# Comparing the Efficacy of 2 WeChat Mini Programs in Reducing Nonmarital Heterosexual Contact by Male Factory Workers: Randomized Controlled Trial

**DOI:** 10.2196/49362

**Published:** 2024-09-09

**Authors:** Kechun Zhang, Bolin Cao, Yuan Fang, Xue Liang, Danhua Ye, Ya Qi Chen, Ruilan Zhong, He Cao, Tian Hu, Ting Li, Yong Cai, Huachun Zou, Zixin Wang

**Affiliations:** 1 Longhua District Center for Disease Control and Prevention Shenzhen China; 2 School of Media and Communication, Shenzhen University Shenzhen China; 3 Department of Health and Physical Education, The Education University of Hong Kong Hong Kong China (Hong Kong); 4 Centre for Health Behaviours Research, The Jockey Club School of Public Health and Primary Care, The Chinese University of Hong Kong Hong Kong China (Hong Kong); 5 Public Health Department, Hongqiao International Institute of Medicine, Tongren Hospital, Shanghai Jiao Tong University School of Medicine Shanghai China; 6 School of Public Health, Fudan University Shanghai China

**Keywords:** nonmarital heterosexual contacts, male factory workers, WeChat mini program, randomized controlled trial, China, mobile phone

## Abstract

**Background:**

Male factory workers in China are vulnerable to HIV transmission. Commercial and nonmarital noncommercial contacts are the driving forces of heterosexual HIV transmission among male factory workers in China. There is a lack of effective HIV interventions for male factory workers in China.

**Objective:**

The primary objective of this randomized controlled trial was to compare the efficacy of an enhanced versus the standard version of a WeChat mini program in reducing sexual intercourse with nonregular female sex partners and female sex workers among male factory workers in Shenzhen, China.

**Methods:**

A nonblinded 2-arm parallel randomized controlled trial was conducted between December 2021 and April 2023. Participants were adult male factory workers in Shenzhen who had access to a smartphone and WeChat. Those who had oral or anal sex with a man or self-reported as HIV positive were excluded. A total of 247 participants were randomly assigned to the intervention group (n=125, 50.6%) or the control group (n=122, 49.4%); 221 (89.5%) and 220 (89.1%) completed follow-up surveys at T1 (6 months after completion of the interventions) and T2 (6 months after T1). Participants in the control group had access to the standard WeChat mini program that provided basic HIV-related knowledge and information about local free HIV testing services. Participants in the intervention group had access to the enhanced WeChat mini program. The enhanced mini program covered all the information in the standard mini program. In addition, the enhanced mini program assessed users’ behaviors and invited users to watch different web-based videos on reducing nonmarital sexual contacts and promoting HIV testing based on their behavioral characteristics at months 0 and 1. The videos were developed based on in-depth interviews with male factory workers. Intention-to-treat analysis was used for outcome analyses. Multiple imputation was used to replace missing outcome values at T1 and T2.

**Results:**

At T1, fewer participants in the intervention group reported sexual intercourse with a nonregular female sex partner in the past 6 months compared with the control group (1/125, 0.8% vs 8/122, 6.6%; relative risk=0.12, 95% CI 0.02-0.96; *P*=.02). However, there were no between-group differences in sexual intercourse with a nonregular female sex partner at T2 (10/125, 8% vs 14/122, 11.5%; *P*=.36) or sexual intercourse with a female sex worker at T1 (2/125, 1.6% vs 2/122, 1.6%; *P*=.98) or T2 (8/125, 6.4% vs 8/122, 6.6%; *P*=.96).

**Conclusions:**

The enhanced WeChat mini program was more effective than the standard WeChat mini program in reducing sexual intercourse with nonregular female sex partners among male factory workers in the short term but not in the longer term. Improvements should be made to the WeChat mini program before implementation.

**Trial Registration:**

ClinicalTrials.gov NCT05811611; https://clinicaltrials.gov/study/NCT05811611

## Introduction

### Overall Situation of HIV Transmission in China

Worldwide, there were 1.5 million new HIV infections and 7.1 to 82 million other new sexually transmitted infection (STI) cases in 2020 [1,2]. In China, the HIV and STI burdens have been increasing over the last few decades [3]. As of 2020, more than 1 million people are living with HIV, and heterosexual contacts continue to be the main transmission route of HIV and other STIs in China; 69.9% and 30.6% to 70% of new HIV and other STI cases, respectively, have been attributed to heterosexual contacts [4]. A sex-specific analysis indicated that the prevalence and mortality rate of HIV in the male population were 2.9 and 3.5 times higher than those in the female population in China, respectively [5]. Factory workers in China, especially male factory workers, are vulnerable to HIV transmission [6]. In recent years, commercial and nonmarital noncommercial contacts, the driving forces of heterosexual HIV transmission in China, have been prevalent among male factory workers [7]. A recent survey of 1361 male factory workers in Shenzhen showed that 24.5% and 21.2% of them had had sexual intercourse with nonregular female sex partners and female sex workers, respectively, in the previous 6 months [7]. Approximately 80% of new HIV cases reported between 2016 and 2018 were among male migrant workers [8].

### HIV-Related Interventions Implemented in China

Sustained efforts have been made by the Chinese government to enhance HIV treatment and care. Recently, the treatment coverage exceeded 90% for people living with HIV in China, along with a treatment success rate of >90% [5]. Regarding HIV risk reduction interventions, most efforts were focused on men who have sex with men and female sex workers. A large number of risk reduction interventions aimed to improve HIV-related knowledge, promote consistent condom use during anal intercourse, increase HIV testing coverage, and implement pre-exposure prophylaxis among men who have sex with men [9-13]. Systematic reviews and meta-analyses have found positive impacts of these interventions in reducing sexual risk behaviors and improving HIV testing uptake [9]. HIV risk reduction interventions targeting female sex workers mainly focus on improving consistent condom use with male clients and HIV testing uptake. A systematic review and meta-analysis of >120 studies in China found that these interventions could effectively improve consistent condom use and HIV testing uptake among female sex workers [14]. However, relatively few HIV risk reduction interventions targeted factory workers [6]. There were only 2 HIV interventions targeting female workers instead of male factory workers, who are at higher risk of HIV in China [15,16]. Although studies have shown that eHealth HIV interventions could reach more participants with low cost and can significantly reduce sexual risk behaviors and increase HIV testing among high-risk groups [17,18], no studies have applied eHealth HIV interventions for factory workers [6]. Therefore, it is necessary to examine the efficacy of eHealth interventions in reducing sexual risk behaviors among factory workers in China using a more rigorous evaluation design.

WeChat is a popular social media site in China with 800 million active users worldwide. WeChat mini programs are “mini-applications” built within the WeChat platform. WeChat allows third parties to develop such mini programs to provide advanced features to users. Users do not need to download or install these mini programs on their smartphones, they only need to scan a QR code or search to open and run these mini programs within WeChat. These features of WeChat mini programs improve their accessibility and convenience. WeChat mini programs have been increasingly used in health promotion and disease management, such as promoting physical activity [19], breastfeeding [20], preventing falls [21], and managing cancer symptoms [22]. To our knowledge, no studies have evaluated the effectiveness of WeChat mini programs in changing HIV-related behaviors. In 2021, the Longhua District Center for Disease Control and Prevention developed a WeChat mini program for factory workers, which provided HIV-related knowledge and information of HIV testing services. However, the WeChat mini program did not have any components targeting nonmarital heterosexual contacts, which are a key risk factor of HIV acquisition among factory workers. In this study, the research team enhanced the standard WeChat mini program by adding interventions that aimed to reduce nonmarital heterosexual contacts among male factory workers.

### Objectives and Hypothesis

The primary objective of this randomized controlled trial (RCT) was to compare the efficacy of an enhanced version versus the standard version of the WeChat mini program in reducing sexual intercourse with nonregular female sex partners and female sex workers in the previous 6 months at T1 (6 months after completing the interventions) and T2 (6 months after T1) among male factory workers in Shenzhen, China. The secondary objectives were to compare the between-group difference in condomless sex with nonregular female sex partners and female sex workers and HIV testing uptake at T1 and T2. We hypothesized that the enhanced version of the WeChat mini program would be more effective in changing the study outcomes than the standard version.

## Methods

### Study Design

**Figure 1:**
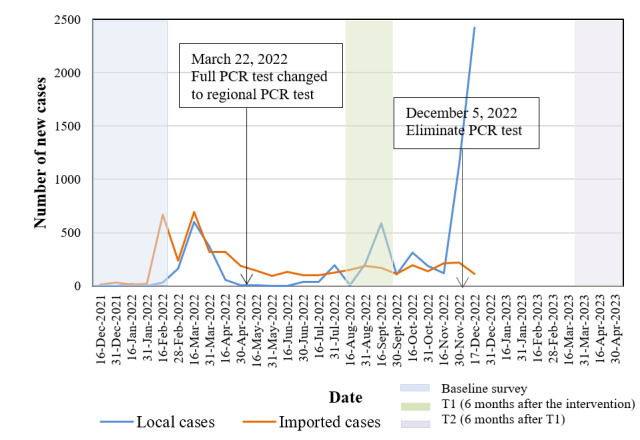
The COVID-19 situation and its control measures in Shenzhen during the study period. There were no official new case data after December 17, 2022. PCR: polymerase chain reaction.

The COVID-19 situation and its control measures in Shenzhen during the study period are presented in Figure 1. We reported this trial in accordance with the CONSORT-EHEALTH (Consolidated Standards of Reporting Trials of Electronic and Mobile Health Applications and Online Telehealth) checklist [23].

### Ethical Considerations

Written informed consent was obtained from all participants. Upon completion of each survey, an e-coupon for ¥50 (US $6.50) was given to the participants as a token of appreciation. All data were stored in the Questionnaire Star (Changsha Ranxing Information Technology Company) server and protected by a password. Only the corresponding author had access to the database. Ethics approval was obtained from the ethics committee of the Longhua District Center for Disease Control and Prevention (reference 2021015).

### Participants and Sample Size Planning

The inclusion criteria were (1) male sex at birth; (2) age of ≥18 years; (3) full-time employee of a factory in Shenzhen, China; (4) a smartphone with internet access; (5) having installed or willingness to install WeChat on the smartphone; and (6) willingness to complete follow-up surveys at T1 and T2. The exclusion criteria were (1) having had oral or anal sex with a man, (2) blindness or deafness, (3) inability to communicate effectively with project staff, and (4) self-report of being HIV positive. Previous studies have shown that approximately 25% of male factory workers in Shenzhen had sexual intercourse with nonregular female sex partners in the previous 6 months [7]. For planning purposes, we assumed that 25% of those in the control group would have had sexual intercourse with nonregular female sex partners or female sex workers in the previous 6 months at T1 or T2. We used a smallest detectable difference of 15% between the intervention and control groups (ie, 10% in the intervention group). Assuming the dropout rate to be 20% at T2, a total of 122 participants per group was required. The total sample size was 244 (PASS; version 11.0; NCSS LLC).

### Recruitment Procedures

In a cross-sectional survey conducted between October 2019 and December 2019, the research team randomly selected 16 factories in Shenzhen from the most up-to-date registry kept by the Longhua District Center for Disease Control and Prevention (approximately 2000 factories in total) to investigate HIV-related behaviors among factory workers. These factories included 4 machinery processing plants, 3 electronic equipment manufacturers, 3 printing and dyeing plants, 2 chemical raw material factories, 1 smelter, 1 clothing factory, 1 food and beverage factory, and 1 other factory. Details of the sampling methods have been reported in published studies [7,24]. The research team obtained the consent of the owners of these 16 factories to recruit participants for this RCT. Since the COVID-19 outbreak, the Shenzhen government requested that each factory establish WeChat groups including all employees to facilitate COVID-19 control [25-27]. During the recruitment period, a coordinator posted the study information in the WeChat groups involving all employers of a participating factory. Interested workers could approach a recruitment booth set by field-workers in the factory. On-site, the field-workers briefed prospective participants about the study procedures and confirmed their eligibility. The field-workers also guaranteed anonymity and participants’ right to withdraw from the study at any time, and refusals would have no consequences. Written informed consent was obtained. We developed web-based baseline and follow-up questionnaires using Questionnaire Star, a commonly used web-based survey platform in China. A link to access a web-based questionnaire was sent to participants’ WeChat at baseline, T1, and T2. Each individual WeChat account was allowed to access the questionnaire once to avoid duplicate responses. The baseline survey contained 60 items (approximately 15 items per page for 4 pages) and required approximately 15 minutes to complete. The follow-up surveys at T1 and T2 had 45 items (approximately 15 items per page for 3 pages) and took approximately 10 minutes to complete. The Questionnaire Star tool performed completeness checks before the questionnaires were submitted. Participants could review and change their responses using the Back button.

Of the 458 participants who visited the recruitment booth during the recruitment period, 398 (86.9%) were eligible for participation after screening, 151 (33%) refused to participate in the study for time or other logistic reasons, and 247 (53.9%) completed the baseline survey. The response rate was 62.1% (247/398). The proportion of participants who completed the follow-up surveys was 89.5% (221/247) at T1 and 89.1% (220/247) at T2.

### The Random Allocation Process

On-site at the recruitment booth, participants were randomized evenly to either the intervention group or the control group. Computer-generated random allocation codes were produced and sealed in opaque envelopes by a research staff member with no involvement in recruitment or the baseline survey. One envelope was drawn and opened in front of the participants by the field-workers. Participants were then informed by the field-workers of which group they were assigned to. Block randomization with block size of 8 was used.

### Development of the Intervention Materials

#### Video Promoting Refusal to Engage in Sexual Behaviors With Nonregular Female Sex Partners and Female Sex Workers

The intervention mainly used an audiovisual approach, which has been used effectively in many HIV-related health promotion programs [12,13,28]. Previous studies have suggested that male factory workers might engage in hazardous sexual behaviors or seek commercial sex under peer pressure [29]. Such findings were confirmed by our previous study among male factory workers in Shenzhen [7]. Hard-to-refuse personal invitations from peers to have sex with nonregular female sex partners or female sex workers was a risk factor of such sexual risk behaviors among male factory workers [7]. Therefore, we developed a web-based video that aimed to increase male factory workers’ capacity to refuse peers’ invitations to have sex with nonregular female sex partners and female sex workers. To inform the contents of the video, in-depth interviews were conducted to understand male factory workers’ perspective of accepting or refusing peers’ invitations to have sex with nonregular female sex partners or female sex workers. In total, 5 male factory workers were recruited through purposive sampling for the in-depth interviews. Before the interviews, field-workers explained the purpose and nature of the interviews. With written informed consent, face-to-face interviews were conducted in private places in the Longhua District Center for Disease Control and Prevention and audio recorded. The interviews lasted approximately 1 hour and were transcribed. A codebook was kept to record special data and transform the data into categories to identify main themes. All informants had been invited by peers to have sex with nonregular female sex partners or female sex workers in the past, and 60% (3/5) of them had had sex with such partners. Four themes were identified as reasons to refuse such invitations: (1) perceiving a high risk of contracting HIV or other STIs through sexual behavior with nonregular female sex partners or female sex workers, (2) female individuals with HIV or STIs might not have any symptoms (look “clean”), (3) HIV or STI infection would have severe consequences on family relationships (their wives or girlfriends would break up with them, and their children or parents would be disappointed regarding their behaviors), and (4) concerns about transmitting HIV or STIs to their wives or stable girlfriends. On the basis of the qualitative findings, a panel consisting of 2 factory worker volunteers, 1 staff member of the Longhua District Center for Disease Control and Prevention, 1 behavioral health expert, and 2 health communication experts was formed to design the health communication messages in the video.

#### Video Promoting HIV Testing Uptake

Another video was developed to promote free HIV testing services provided by the Longhua District Center for Disease Control and Prevention. The research team conducted a survey investigating facilitators and barriers to uptake of HIV testing among male factory workers who had sexual intercourse with nonregular female sex partners or female sex workers [24]. Belief that taking up HIV testing could detect HIV infection earlier to enable better treatment outcomes was a facilitator for these factory workers to undergo HIV testing. Concerns about being stigmatized and inconvenience were found to be barriers [24]. The aforementioned in-depth interviews of male factory workers confirmed these findings. The contents of the video addressed these facilitators and barriers. Both videos were produced by a professional team, reviewed by 5 other male factory workers, and finalized by the panel.

### The Control Group

**Figure 2:**
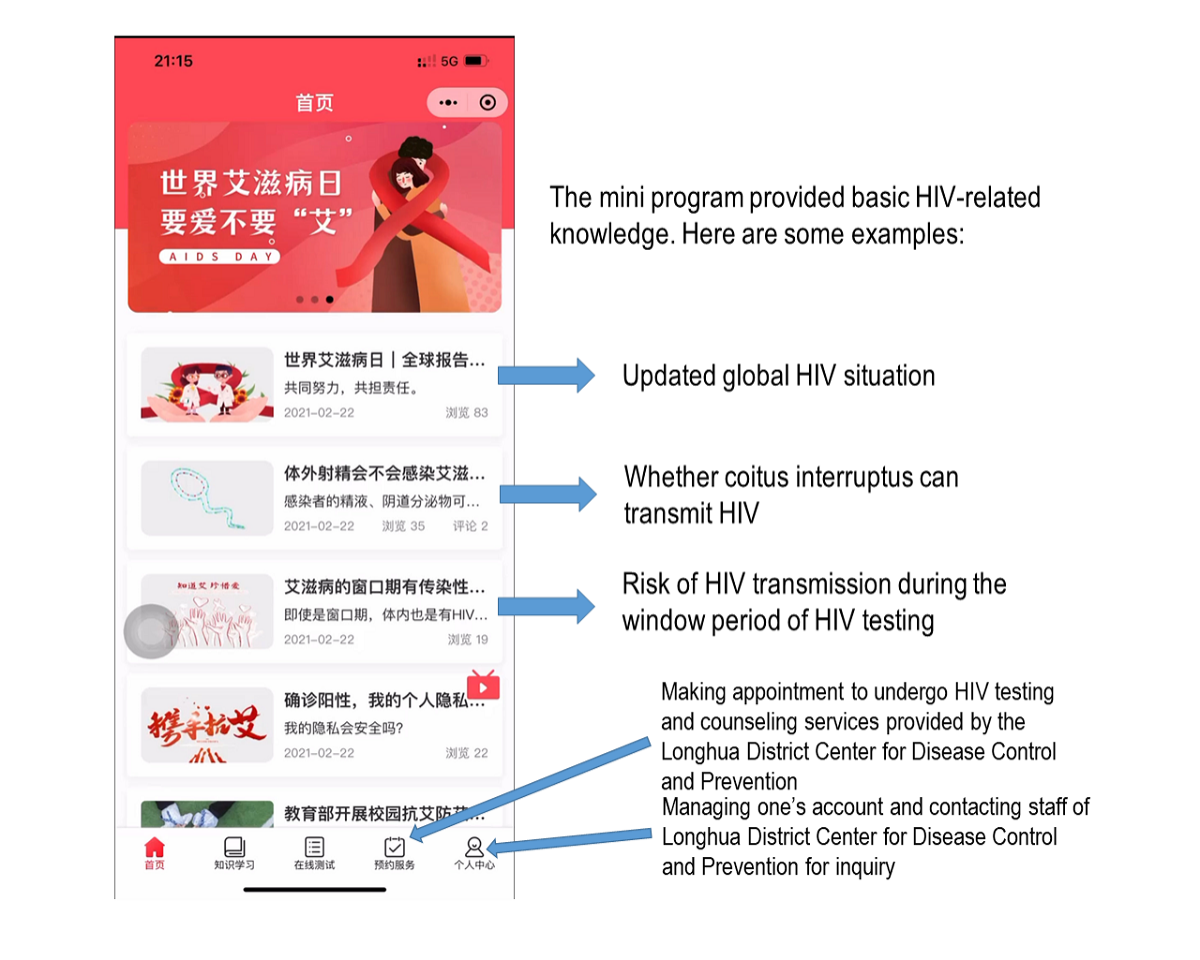
Standard version of the WeChat mini program developed by the Longhua District Center for Disease Control and Prevention (control group).

Participants scanned a QR code to run the standard version of the WeChat mini program developed by the Longhua District Center for Disease Control and Prevention. The mini program provided basic HIV-related knowledge, including (1) what are HIV and AIDS, (2) how is HIV spread, (3) how can a person reduce the risk of contracting HIV, (4) what is the treatment for HIV, and (5) what are the symptoms of HIV and AIDS. Users could also access information about free HIV testing and counseling services provided by the Longhua District Center for Disease Control and Prevention and make an appointment for HIV testing. In addition, they could contact the staff of Longhua District Center for Disease Control and Prevention for inquiry through private messaging. Figure 2 shows the standard version of the WeChat mini program in the control group.

### The Intervention Group

#### Overview

**Figure 3:**
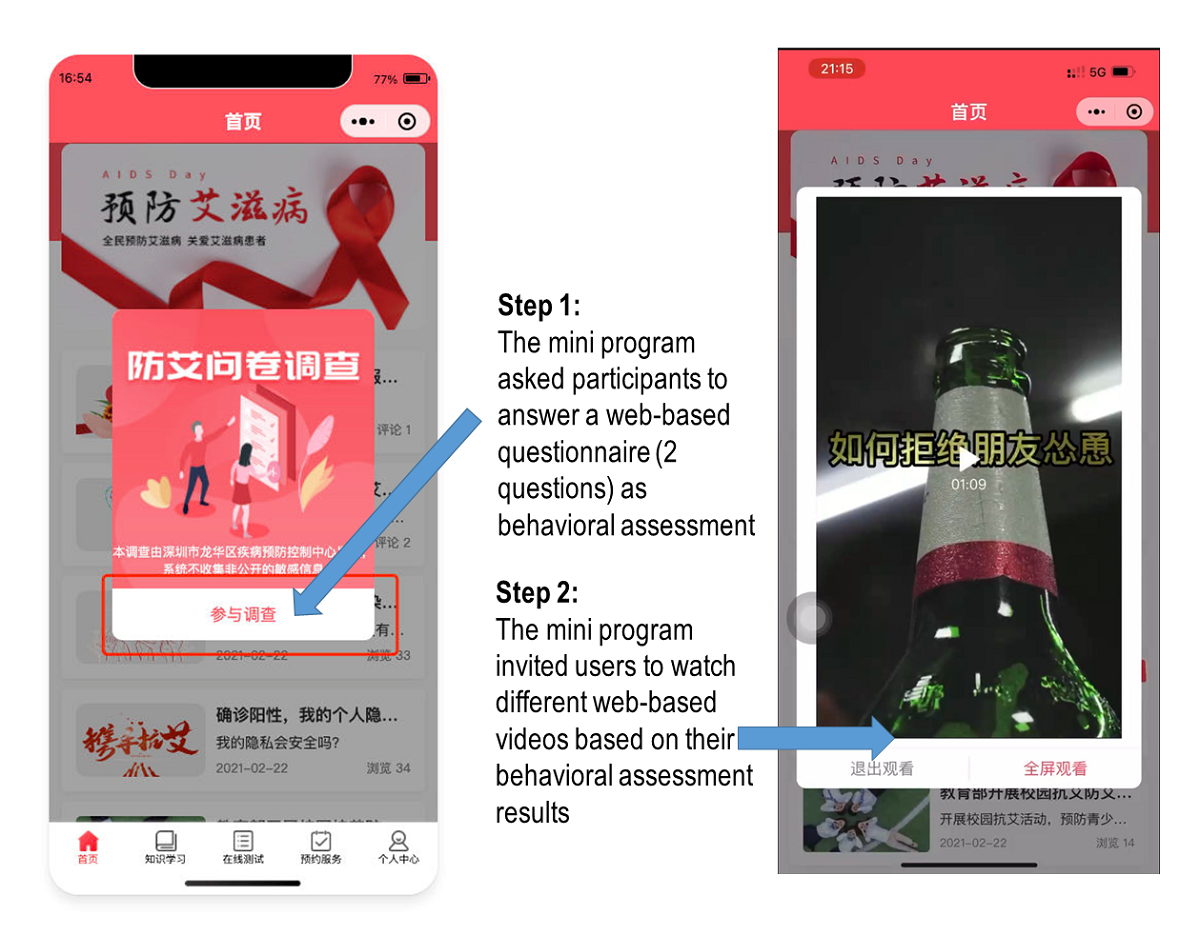
Enhanced version of the WeChat mini program developed by the Longhua District Center for Disease Control and Prevention (intervention group).

Participants scanned another QR code to run the enhanced WeChat mini program. The enhanced mini program covered all the information in the standard version of the WeChat mini program developed by the Longhua District Center for Disease Control and Prevention. In addition, the enhanced mini program assessed users’ behaviors and invited users to watch different videos based on their behavioral characteristics at months 0 and 1. To avoid participants watching the same video twice, 2 slightly different versions of each video were prepared. In each session, the mini program randomly selected 1 version for the participants. Figure 3 shows the demonstration of the enhanced version of the WeChat mini program in the intervention group.

#### Behavioral Assessment

The mini program asked participants to answer two questions: (1) whether they had had sex with nonregular female sex partners or female sex workers in the previous month and (2) whether they intended to have sex with nonregular female sex partners or female sex workers in the following month. Participants chose their response on the screen (yes or no). If the participant answered “yes” to at least one question, he was classified as “higher risk” by the mini program. If the participant answered “no” to both questions, he was considered as “lower risk.” Answering these questions was mandatory.

#### Video for People at Lower Risk

A video popped up on the screen to promote refusal to peers’ invitations to engage in sexual behaviors with nonregular female sex partners and female sex workers. In the video, a male factory worker was trying to persuade his friend not to have sex with nonregular female sex partners and female sex workers. He emphasized that (1) female individuals with HIV might not have any symptoms, (2) there is a high risk of HIV acquisition through sexual behavior with nonregular female sex partners or female sex workers, (3) there are severe consequences of HIV infection for family relationships (breaking up with his wife, and his children or parents being disappointed regarding his behaviors), and (4) there was a high risk of transmitting HIV to his wife.

#### Video for People at Higher Risk

In addition to the video watched by people at lower risk, another video popped up on the screen to promote HIV testing. The contents included (1) a male factory worker introducing the benefits of taking up HIV testing, such as that it would help detect HIV infection earlier and reduce psychological burden; and (2) the same factory worker demonstrating the procedures of free HIV testing and counseling at the Longhua District Center for Disease Control and Prevention, which portrayed caring, supportive, and nonjudgmental administrators regardless of users’ test results.

### Fidelity Assessments

To ensure a complete exposure, the videos were formatted in such a way that the participants could not fast-forward or skip any part of them. The mini program automatically documented the number of sessions completed by participants in the intervention group.

### Measures to Avoid Contamination

The videos in the enhanced version of the WeChat mini program could not be downloaded or forwarded to other people. Participants in the intervention group were asked not to disseminate the QR code to access the enhanced mini program to other people. The research team also cross-checked the user IDs of the standard and the enhanced mini programs during the project period.

### Outcome Measurements

Sexual intercourse with nonregular female sex partners and female sex workers in the previous 6 months were the primary outcomes. In total, 2 questions developed by published studies measured these behaviors at baseline, T1, and T2 (ie, “Did you have sexual intercourse with a nonregular female sex partner in the past 6 months?” and “Did you have sexual intercourse with a female sex worker in the past 6 months?”). Similarly to the published studies, a female sex worker was defined as a woman who explicitly exchanged sex for money or gifts, and a nonregular female sex partner was defined as a woman who was neither a female sex worker nor their regular female sex partner (stable girlfriend or wife) [7,24].

Secondary outcomes measured at baseline, T1, and T2 included (1) condomless sex with a nonregular female sex partner among participants who had sexual intercourse with a nonregular female sex partner in the previous 6 months, (2) condomless sex with a female sex worker among participants who had sexual intercourse with a female sex worker in the previous 6 months, and (3) uptake of any type of HIV testing among all participants in the previous 6 months. These secondary outcomes were also measured using validated questions [7,24].

Background characteristics were collected through a baseline survey, including sociodemographics and use of HIV or STI prevention services other than HIV testing.

### Process Evaluation

At T1, participants in the intervention group were asked whether the contents of the videos were interesting, relevant to them, informative, helpful, valuable, persuasive, and credible. They were also asked whether they were willing to share these videos with their peers. The response categories of the process evaluation questions were 1=*strongly disagree*, 2=*disagree*, 3=*neutral*, 4=*agree*, and 5=*strongly agree*. In addition, COVID-19 vaccination status and history of confirmed COVID-19 infection were measured at both T1 and T2.

### Statistical Analysis

Between-group differences in baseline characteristics were compared using chi-square tests. The baseline characteristics of participants who completed the survey at T1 or T2 and those who were lost to follow-up were also compared using chi-square tests. Intention-to-treat analysis was used for the outcome analyses. Missing data were all binary variables. As there were no missing data among participants who completed the T1 and T2 surveys, the missing rate was equal to the dropout rate. We filled the missing outcome values at T1 or T2 using multiple imputations. The Markov chain Monte Carlo method was used to impute data with an arbitrary pattern of missing values, whereas monotone methods were used to impute data with a monotone pattern of missing values. Predictors included baseline background characteristics and baseline values of these outcomes. The relative risk, absolute risk reduction, and number needed to treat and their 95% CIs were calculated using Microsoft Excel (Microsoft Corp). Logistic regression models were used to test the between-group difference in the imputed primary and secondary outcomes after controlling for baseline background characteristics, with *P*<.05 for between-group comparisons. Crude odds ratios, adjusted odds ratios, and their 95% CIs were obtained. As relationship status might affect the onset of sexual risk behaviors, subgroup analyses of the primary and secondary outcomes were conducted among participants with and without a stable girlfriend or spouse at baseline. Within-group changes in the imputed primary and secondary outcomes (month 6 vs baseline) were investigated using McNemar tests. SPSS (version 26.0; IBM Corp) was used for data analysis, and *P*<.05 was considered statistically significant.

## Results

### Descriptive Statistics

**Figure 4:**
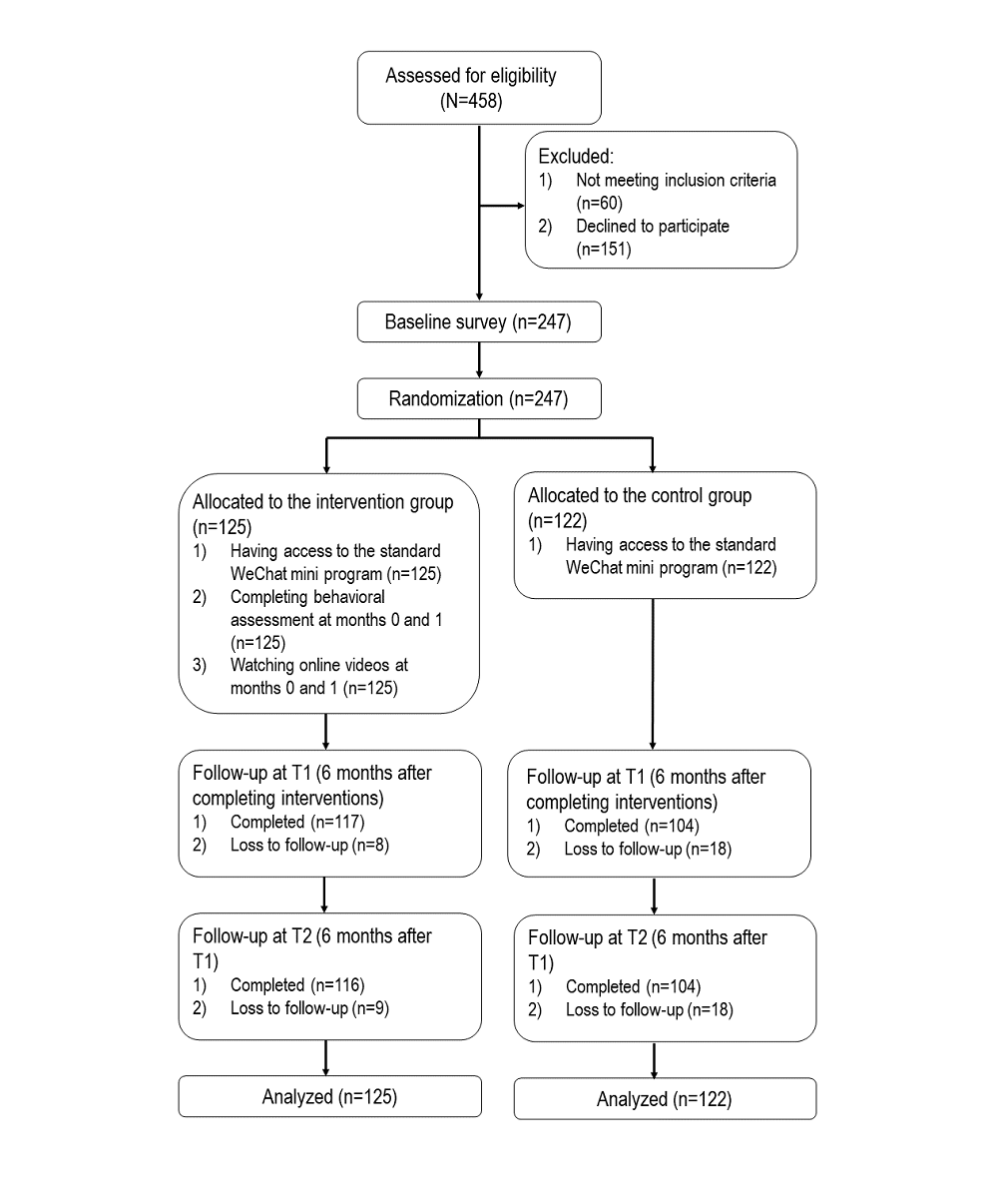
The CONSORT (Consolidated Standards of Reporting Trials) flowchart of the randomized controlled trial.

A nonblinded 2-arm parallel RCT was conducted between December 2021 and April 2023. The trial was registered at ClinicalTrials.gov (NCT05811611). The CONSORT (Consolidated Standards of Reporting Trials) flowchart is shown in Figure 4. At baseline, over half of the participants were aged ≤40 years (204/247, 82.6%), were internal migrants (226/247, 91.5%), were married to a woman or had a stable girlfriend (173/247, 70%), had attained tertiary education (135/247, 54.7%), had a monthly income of ¥5000-9999 (US $782.9-$1564.8; 151/247, 61.1%), lived with their partner or children in Shenzhen (139/247, 56.3%), and were frontline workers (167/247, 67.6%). In the previous 6 months, 36.4% (90/247) had had sexual intercourse with a nonregular female sex partner, and 62% (56/90) of those with a nonregular female sex partner reported condomless sex with such a partner. Of 86 participants (86/247, 34.8% of all participants) who had sexual intercourse with a female sex worker, 64 (74%) reported condomless sex with such a partner. Among the participants, 4.9% (12/247) had used HIV testing, and 19.8% (49/247) had used other HIV or STI prevention services in the previous 6 months. No significant between-group differences in these baseline characteristics were found (*P* values between .07 and .97; Table 1).

The dropout rate in the intervention group and the control group was 6.4% (8/125) and 14.8% (18/122) at T1 and 7.2% (9/125) and 14.8% (18/122) at T2, respectively. In the intervention group, participants with a lower educational level and those who were internal migrants in the control group were more likely to drop out at both T1 and T2 (Multimedia Appendices 1 and 2).

**Table 1:** Characteristics of the participants at baseline (N=247).

Characteristic				All, n (%)		Intervention group (n=125), n (%)		Control group (n=122), n (%)		*P* value	
**Sociodemographic characteristics**											
	**Age group (y)**										.22
		18-30	92 (37.2)		47 (37.6)		45 (36.9)				
		31-40	112 (45.3)		62 (49.6)		50 (41)				
		41-50	33 (13.4)		13 (10.4)		20 (16.4)				
		>50	10 (4)		3 (2.4)		7 (5.7)				
	**Ethnicity**										.57
		Han	231 (93.5)		118 (94.4)		113 (92.6)				
		Minority	16 (6.5)		7 (5.6)		9 (7.4)				
	**Registered permanent resident of Shenzhen**										.12
		No	226 (91.5)		111 (88.8)		115 (94.3)				
		Yes	21 (8.5)		14 (11.2)		7 (5.7)				
	**Relationship status**										.32
		Currently single	74 (30)		41 (32.8)		33 (27)				
		Stable girlfriend or married to a woman	173 (70)		84 (67.2)		89 (73)				
	**Educational level**										.72
		Junior high or lower	50 (20.2)		23 (18.4)		27 (22.1)				
		Senior high or equivalent	62 (25.1)		31 (24.8)		31 (25.4)				
		College and higher	135 (54.7)		71 (56.8)		64 (52.5)				
	**Living with partner, spouse, or children in Shenzhen**										.73
		No	108 (43.7)		56 (44.8)		52 (42.6)				
		Yes	139 (56.3)		69 (55.2)		70 (57.4)				
	**Monthly personal income**										.07
		<¥3000 (US $469.5)	12 (4.9)		9 (7.2)		3 (2.5)				
		¥3000-¥4999 (US $469.5-$782.3)	69 (27.9)		31 (24.8)		38 (31.1)				
		¥5000-¥9999 (US $782.9-$1564.8)	151 (61.1)		74 (59.2)		77 (63.1)				
		≥¥10,000 (US $1564.9)	15 (6.1)		11 (8.8)		4 (3.3)				
	**Status as frontline worker or management staff**										.49
		Frontline worker	167 (67.6)		82 (65.6)		85 (69.7)				
		Management staff	80 (32.4)		43 (34.4)		37 (30.3)				
**HIV or STI^a^ prevention service use in the previous 6 months**											
	**Use of HIV testing**										.97
		No	235 (95.1)		119 (95.2)		116 (95.1)				
		Yes	12 (4.9)		6 (4.8)		6 (4.9)				
	**Use of other HIV or STI prevention services (receiving free condoms or pamphlets or attending workshops or seminars)**										.10
		No	198 (80.2)		95 (76)		103 (84.4)				
		Yes	49 (19.8)		30 (24)		19 (15.6)				
**Sexual behaviors in the previous 6 months**											
	**Sexual intercourse with nonregular female sex partners**										.70
		No	157 (63.6)		78 (62.4)		79 (64.8)				
		Yes	90 (36.4)		47 (37.6)		43 (35.2)				
	**Sexual intercourse with female sex workers**										.23
		No	161 (65.2)		77 (61.6)		84 (68.9)				
		Yes	86 (34.8)		48 (38.4)		38 (31.1)				
	**Condomless sex with nonregular female sex partners (among participants who had sexual intercourse with nonregular female sex partners in the previous 6 months)**										.74
		No	34 (37.8)^b^		17 (36.2)^c^		17 (39.5)^d^				
		Yes	56 (62.2)^b^		30 (63.8)^c^		26 (60.5)^d^				
	**Condomless sex with female sex workers (among participants who had sexual intercourse with female sex workers in the previous 6 months)**										.39
		No	22 (25.6)^e^		14 (29.2)^f^		8 (21.1)^g^				
		Yes	64 (74.4)^e^		34 (70.8)^f^		30 (78.9)^g^				

^a^STI: sexually transmitted infection.

^b^n=90.

^c^n=47.

^d^n=43.

^e^n=86.

^f^n=48.

^g^n=38.

### Primary Outcomes

At T1, fewer participants in the intervention group reported sexual intercourse with a nonregular female sex partner in the previous 6 months as compared with those in the control group (1/125, 0.8% vs 8/122, 6.6%; relative risk=0.12, 95% CI 0.02-0.96; absolute risk reduction=–0.06, 95% CI –0.10 to –0.01; number needed to treat=–17.4, 95% CI –91.3 to –9.6; *P*=.02). However, there were no between-group differences in sexual intercourse with a nonregular female sex partner at T2 (10/125, 8% vs 14/122, 11.5%; *P*=.36) or sexual intercourse with a female sex worker at T1 (2/125, 1.6% vs 2/122, 1.6%; *P*=.98) or T2 (8/125, 6.4% vs 8/122, 6.6%; *P*=.96). There was no between-group difference in these primary outcomes among the subgroups of participants with and without a stable girlfriend or spouse at baseline (Table 2).

When comparing data measured at T1 or T2 with the baseline data, significant decreases in sexual intercourse with a nonregular female sex partner and with a female sex worker were observed in both the intervention group and the control group (*P*<.001 in all comparisons; Table 3). As compared with T1, significant increases in sexual intercourse with a nonregular female sex partner (*P*=.01) and with a female sex worker (*P*=.03) were observed in the intervention group at T2. A significant increase in sexual intercourse with a female sex worker was observed when comparing T2 versus T1 among participants in the control group (*P*=.03; Table 3).

**Table 2:** Between-group differences in the primary and secondary outcomes.

					Intervention group, n (%)		Control group, n (%)		RR^a^ (95% CI)		ARR^b^ (95% CI)		NNT^c^ (95% CI)		*P* value
**Primary outcomes**															
	**Sexual intercourse with nonregular female sex partners**														
		**All participants**													
			T1^d^	1 (0.8)^e^		8 (6.6)^f^		0.12 (0.02 to 0.96)		–0.06 (–0.10 to –0.01)		–17.4 (–91.3 to –9.6)		.02	
			T2^g^	10 (8)^e^		14 (11.5)^f^		0.70 (0.32 to 1.51)		–0.03 (–0.11 to 0.04)		–28.8 (–9.2 to 25.5)		.36	
		**Subgroup of participants without a stable girlfriend or spouse at baseline**													
			T1	0 (0)^h^		2 (6.1)^i^		—^j^		–0.06 (–0.14 to 0.02)		–16.5 (–7.0 to 48.1)		.20	
			T2	5 (12.2)^h^		6 (18.2)^i^		0.68 (0.22 to 2.00)		–0.06 (–0.22 to 0.10)		–16.7 (–4.43 to 9.48)		.53	
		**Subgroup of participants with a stable girlfriend or spouse at baseline**													
			T1	1 (1.2)^k^		6 (6.7)^l^		0.18 (0.02 to 1.44)		–0.06 (–0.11 to 0.001)		–18.1 (–8.9 to 661.3)		.07	
			T2	5 (6)^k^		8 (9)^l^		0.59 (0.21 to 1.69)		–0.03 (–0.11 to 0.05)		–32.9 (–9.22 to 20.97)		.54	
	**Sexual intercourse with female sex workers**														
		**All participants**													
			T1	2 (1.6)^e^		2 (1.6)^f^		0.98 (0.14 to 6.82)		0.0 (–0.03 to 0.03)		2541.7 (–31.4 to 32.2)		.98	
			T2	8 (6.4)^e^		8 (6.6)^f^		0.98 (0.38 to 2.51)		–0.02 (–0.06 to 0.06)		635.4 (–15.9 to 16.7)		.96	
		**Subgroup of participants without a stable girlfriend or spouse at baseline**													
			T1	0 (0)^h^		1 (3)^i^		—		–0.03 (–0.09 to 0.03)		–33.0 (–11.26 to 35.48)		.45	
			T2	4 (9.8)^h^		4 (12.1)^i^		0.80 (0.22 to 2.98)		–0.02 (–0.17 to 0.12)		–42.28 (–5.98 to 8.33)		>.99	
		**Subgroup of participants with a stable girlfriend or spouse at baseline**													
			T1	2 (2.4)^k^		1 (1.1)^l^		2.11 (0.20 to 22.93)		0.01 (–0.03 to 0.05)		79.53 (–37.45 to 19.28)		.48	
			T2	4 (4.8)^k^		4 (4.5)^l^		1.06 (0.27 to 4.10)		0.002 (–0.06 to 0.06)		373.8 (–16.67 to 15.30)		>.99	
**Secondary outcomes**															
	**Condomless sex with nonregular female sex partners (among participants who had sexual intercourse with a nonregular female sex partner at each time point)**														
		**All participants**													
			T1	0 (0)^m^		5 (62.5)^n^		—		0.63 (–0.96 to –0.29)		–1.6 (–3.5 to –1.0)		.24	
			T2	6 (60)^o^		3 (21.4)^p^		2.80 (0.91 to 8.61)		0.39 (0.01 to 0.76)		2.59 (1.32 to 72.99)		.05	
		**Subgroup of participants without a stable girlfriend or spouse at baseline**													
			T1	0 (0)^q^		1 (50)^r^		—		—		—		—	
			T2	2 (40)^s^		1 (16.7)^t^		2.40 (0.30 to 19.33)		0.23 (–0.29 to 0.76)		4.29 (–3.55 to 1.32)		.39	
		**Subgroup of participants with a stable girlfriend or spouse at baseline**													
			T1	0 (0)^m^		4 (66.7)^t^		—		–0.67 (–1.04 to –0.29)		–1.5 (–0.35 to –0.96)		.21	
			T2	4 (80)^s^		2 (25)^n^		3.20 (0.89 to 11.48)		0.55 (0.09 to 1.01)		1.82 (0.99 to 11.30)		.05	
	**Condomless sex with female sex workers (among participants who had sexual intercourse with a female sex worker at each time point)**														
		**All participants**													
			T1	0 (0)^r^		0 (0)^r^		—		—		—		—	
			T2	0 (0)^n^		0 (0)^n^		—		—		—		—	
		**Subgroup of participants without a stable girlfriend or spouse at baseline**													
			T1	0 (0)^q^		0 (0)^m^		—		—		—		—	
			T2	0 (0)^u^		0 (0)^u^		—		—		—		—	
		**Subgroup of participants with a stable girlfriend or spouse at baseline**													
			T1	0 (0)^r^		0 (0)^m^		—		—		—		—	
			T2	0 (0)^u^		0 (0)^u^		—		—		—		—	
	**Uptake of any type of HIV testing**														
		**All participants**													
			T1	10 (8)^e^		10 (8.2)^f^		0.98 (0.42 to 2.26)		–0.002 (–0.07 to 0.07)		–508.3 (–14.3 to 15.1)		.57	
			T2	10 (8)^e^		11 (9)^f^		0.89 (0.39 to 2.01)		–0.01 (–0.08 to 0.05)		–98.4 (–12.5 to 16.8)		.47	
		**Subgroup of participants without a stable girlfriend or spouse at baseline**													
			T1	3 (7.3)^h^		2 (6.1)^i^		1.21 (0.21 to 6.81)		0.01 (–0.10 to 0.13)		79.59 (–9.86 to 7.91)		.83	
			T2	2 (4.9)^h^		2 (6.1)^i^		0.80 (0.12 to 5.41)		–0.01 (–0.12 to 0.09)		–84.56 (–8.58 to 10.76)		.82	
		**Subgroup of participants with a stable girlfriend or spouse at baseline**													
			T1	7 (8.3)^k^		8 (9)^l^		0.82 (0.32 to 2.11)		–0.01 (–0.09 to 0.08)		–152.57 (–11.07 to 12.94)		.88	
			T2	8 (9.5)^k^		9 (10.1)^l^		0.94 (0.38 to 2.33)		–0.01 (–0.09 to 0.08)		–169.91 (–10.57 to 12.08)		.69	

^a^RR: relative risk.

^b^ARR: absolute risk reduction.

^c^NNT: number needed to treat.

^d^T1: 6 months after completion of the interventions.

^e^n=125.

^f^n=122.

^g^T2: 6 months after T1.

^h^n=41.

^i^n=33.

^j^Not applicable.

^k^n=84.

^l^n=89.

^m^n=1.

^n^n=8.

^o^n=10.

^p^n=14.

^q^n=0.

^r^n=2.

^s^n=5.

^t^n=6.

^u^n=4.

**Table 3:** Within-group changes in primary and secondary outcomes.

				Intervention group (n=125)		Control group (n=122)
**Primary outcomes**						
	**Sexual intercourse with nonregular female sex partners**					
		Baseline, n (%)	47 (37.6)		43 (35.2)	
		T1^a^, n (%)	1 (0.8)		8 (6.6)	
		T2^b^, n (%)	10 (8)		14 (11.5)	
		T1 vs baseline, *P* value	<.001		<.001	
		T2 vs baseline, *P* value	<.001		<.001	
		T2 vs T1, *P* value	.01		.42	
	**Sexual intercourse with female sex workers**					
		Baseline, n (%)	48 (38.4)		38 (31.1)	
		T1, n (%)	2 (1.6)		2 (1.6)	
		T2, n (%)	8 (6.4)		8 (6.6)	
		T1 vs baseline, *P* value	<.001		<.001	
		T2 vs baseline, *P* value	<.001		<.001	
		T2 vs T1, *P* value	.03		.03	
**Secondary outcome**						
	**Uptake of any type of HIV testing**					
		Baseline, n (%)	6 (4.8)		6 (4.9)	
		T1, n (%)	10 (8)		10 (8.2)	
		T2, n (%)	10 (8)		11 (9)	
		T1 vs baseline, *P* value	.33		.51	
		T2 vs baseline, *P* value	.33		.34	
		T2 vs T1, *P* value	>.99		.73	

^a^T1: 6 months after completion of the interventions.

^b^T2: 6 months after T1.

### Secondary Outcomes

There was no significant between-group difference in condomless sex with a nonregular female sex partner at T1 or T2. No participants in the intervention group or the control group reported condomless sex with a female sex worker at T1 or T2. Participants in the intervention group and the control group reported similar HIV testing uptake in the previous 6 months at T1 (10/125, 8% vs 10/122, 8.2%; *P*=.57) and T2 (10/125, 8% vs 11/122, 9%; *P*=.47). There was no between-group difference in these secondary outcomes between the subgroups of participants with and without a stable girlfriend or spouse at baseline (Table 2). There were no within-group changes in HIV testing uptake in the intervention group or the control group (Table 3).

### Process Evaluation

As documented by the WeChat mini program, all participants in the intervention group (125/125, 100%) completed a behavioral assessment and watched the videos at months 0 and 1. No participants in the control group accessed the enhanced mini program during the project period. Among the 117 participants in the intervention group who completed the process evaluation at T1, approximately half of them strongly agreed or agreed that contents of the videos were interesting (n=54, 46.2%), relevant to them (n=53, 45.3%), informative (n=53, 45.3%), helpful (n=57, 48.7%), valuable (n=57, 48.7%), persuasive (n=56, 47.9%), and credible (n=56, 47.9%). Moreover, 53% (62/117) of them were willing to share these videos with their peers in the future. At T1, all participants in the intervention group (117/117, 100%) and the control group (104/104, 100%) who completed the follow-up survey received 2 doses of the COVID-19 vaccination. There was no significant between-group difference in the uptake rate of the third dose of the COVID-19 vaccination (110/117, 94% in the intervention group vs 95/104, 91.3% in the control group; *P*=.44). At T2, all participants who completed the follow-up survey received 3 doses of the COVID-19 vaccination (116/116, 100% in the intervention group vs 104/104, 100% in the control group; *P*>.99). No participant reported a history of COVID-19 infection at T1. At T2, a total of 65.5% (76/116) of the participants in the intervention group and 71.2% (74/104) of the participants in the control group reported a history of COVID-19 infection (*P*=.37 for between-group comparison).

## Discussion

### Value and Strengths of This Study

Our study contributed to the literature by comparing the efficacy of an enhanced version versus the standard version of a WeChat mini program using an RCT design and developing a new method to reduce nonmarital heterosexual contacts among male factory workers. Our study also had the strengths of intervention materials developed based on in-depth interviews, a long follow-up duration, and a low dropout rate and was based on the findings of formative studies [7,24].

### Findings of and Elaborations on the Primary Outcomes

As compared with the baseline data, significant decreases in sexual intercourse with nonregular female sex partners and with female sex workers were observed in both the intervention group and the control group at T1 and T2. Our results showed that adding health promotion videos to the standard WeChat mini program increased its efficacy in reducing sexual intercourse with nonregular female sex partners at T1 (1/125, 0.8% vs 8/122, 6.6%). However, there is a lack of long-term efficacy as no between-group differences in sexual intercourse with nonregular female sex partners or female sex workers were observed at T2. The lack of additional interventions (eg, videos, reminders, and notifications) might contribute to the absence of long-term effects on sexual risk behaviors. There are some potential strategies to improve our interventions. First, it is beneficial to have more intervention sessions and a longer intervention period. Our mini program only disseminated health promotion twice 1 month apart. A systematic review and meta-analysis showed that multiple-session interventions were more effective than single-session interventions in improving consistent condom use [14]. An intervention duration of 3 to 6 months is commonly used in successful HIV risk reduction interventions and WeChat mini programs for other health-related behaviors [30-32]. Therefore, future studies may evaluate the effectiveness of larger doses of our interventions (eg, 1 session per month for 3-6 months) in reducing sexual risk behaviors in the longer term. Second, some strategies used by successful WeChat mini programs for other types of health promotion have shed some light regarding improving our interventions. These programs made use of behavior change theories (eg, the Behavior Change Wheel or the Health Action Process Approach theory) and were equipped with real-time question-and-answer functions [31,32]. Interventions based on behavior change theories are more likely to be effective than non–theory-based ones [33]. Providing real-time answers to participants’ individual questions could improve engagement and, hence, improve the effectiveness of digital health interventions [34]. In addition, the process evaluation showed that only half of the participants were satisfied with the additional health promotion videos in the enhanced WeChat mini program. Therefore, it is necessary to improve the contents of these health promotion materials. Similarly to most HIV interventions for factory workers [6], our interventions were developed using a top-down approach in which end users’ involvement was limited. Making use of cocreation, which refers to collaborative public health intervention development by academics together with end users [35-37], may be useful in improving the health promotion materials. Such an approach is considered to be promising and efficient for addressing complex health issues and facilitating behavior change [35-37].

### Findings of and Elaborations on the Secondary Outcome

Regarding the secondary outcomes, no between-group difference in HIV testing was observed at T1 or T2. The HIV testing rate in both groups at T1 and T2 was similar to that in the baseline data. As the prevalence of sexual intercourse with nonregular female sex partners or female sex workers at T1 and T2 was much lower than that in the baseline data, participants might perceive that they were at low risk of HIV infection and, hence, had no need to undergo HIV testing.

### Impact of the COVID-19 Pandemic

The changes in the COVID-19 situation and its control measures had impacts on our study outcomes. At baseline (December 2021 to February 2022), the COVID-19 situation was stable and under control in Shenzhen. Starting from April 2022, the city was continuously hit by a COVID-19 outbreak caused by Omicron variants BA2 or BA5. In response to the outbreak, the city implemented strict control measures, including regular universal COVID-19 testing, territory lockdown, and closure of entertainment premises [38]. The implementation of these control measures increased difficulties for male factory workers to seek nonregular female sex partners or female sex workers. When China started to relieve its COVID-19 control measures in December 2022, the number of new cases increased dramatically. It was estimated that >70% of all people in China were infected with SARS-CoV-2 during the huge wave between December 2022 and February 2023 [39]. It was likely that most of our participants were infected with SARS-CoV-2 during this period. The discomfort and symptoms of COVID-19 might reduce their motivation to have sex with nonregular female sex partners or female sex workers. The aforementioned situation could partially explain why the magnitude of decreases in sexual risk behaviors in our study (T1 vs baseline: 28.6%-36.8%; T2 vs baseline: 24.5%-36.8%) was much larger than those of previous interventions (posttest vs pretest: 3.1%-14%) targeting factory workers [40-44]. Moreover, facility-based HIV testing was seriously affected by the COVID-19 outbreak in China [45-48]. Some governmental HIV testing facilities were suspended during the pandemic as the government reallocated resources to focus on COVID-19 control. The decreased access to facility-based HIV testing might also contribute to the low HIV testing uptake observed in our study.

### Limitations of the Study

Our study also had other limitations. First, although we measured COVID-19 vaccination status and history of confirmed COVID-19 infection at both T1 and T2, we did not ask about the direct impact of the COVID-19 situation on participants’ sexual behaviors. The strict COVID-19 measures implemented in the study site applied to everyone. As this study was an RCT, the impact of the COVID-19 situation on the study outcomes was expected to be similar between the intervention and control groups. Second, we did not have a control group without access to the WeChat mini program. Therefore, it was unclear whether the standard or enhanced WeChat mini program would be more effective than no intervention in reducing sexual risk behaviors among factory workers. Third, similarly to most interventional studies targeting factory workers, our participants were recruited through convenience sampling. Information from factory workers who refused to join this study was unavailable. As compared with a larger and more representative sample of male factory workers in the same city [7], our participants were older (155/247, 62.9% vs 49.1% aged >30 years) and more likely to have a stable girlfriend or be married to a woman (173/247, 70% vs 40.9%), have sexual intercourse with a nonregular female sex partner (90/247, 36.4% vs 24.5%) and with a female sex worker (86/247, 34.8% vs 21.3%), and report condomless sex with a nonregular female sex partner (56/90, 62% vs 21.3%) or with a female sex worker (64/86, 74% vs 13.5%). Therefore, selection bias existed. Our results were more applicable to male factory workers who were older, had a stable partner, and engaged in sexual risk behaviors. Future studies should explore the effectiveness of our interventions in a more representative sample of male factory workers. Fourth, only male factory workers in 1 Chinese city were included in this study. Generalization should be made cautiously to other Chinese cities. Fifth, self-reported responses might cause report bias despite the anonymous nature of the study. Participants might underreport sexual risk behaviors and overreport HIV testing uptake due to social desirability. Verification of these self-reported responses was impossible. Moreover, a long-distance relationship (eg, a stable partner who lives in another city) may have a different impact on the study outcomes as compared with a short-distance relationship (eg, living with a stable partner in the same city). In the baseline survey, we did not measure whether participants who had a stable girlfriend or were married to a woman were living with either their girlfriend or their spouse in Shenzhen. It is possible that some of them were living with their children but not with their girlfriend or spouse. Furthermore, attrition bias might exist. Participants with a lower education level in the intervention group and those who were internal migrants in the control group were more likely to drop out. As educational level and being an internal migrant were not significant factors of sexual intercourse with nonregular female sex partners or female sex workers [7], the impact of attrition on the study outcomes might be limited.

### Conclusions

In summary, the RCT findings showed that the enhanced version of the WeChat mini program with web-based videos addressing risk factors of sexual intercourse with nonregular female sex partners and female sex workers and promoting HIV testing was more effective than the standard version in reducing sexual intercourse with nonregular female sex partners in the short term but not in the longer term. Improvements should be made to the WeChat mini program before implementation.

## Appendix

Multimedia Appendix 1 Comparison of baseline characteristics between participants who completed the follow-up evaluation at T1 (6 months after completion of the intervention) and those who were lost to follow-up.

Multimedia Appendix 2 Comparison of baseline characteristics between participants who completed the follow-up evaluation at T2 (6 months after T1; 6 months after completion of the intervention) and those who were lost to follow-up.

Multimedia Appendix 3 CONSORT-eHEALTH checklist (V 1.6.1).

## Abbreviations

CONSORT: Consolidated Standards of Reporting Trials
CONSORT-EHEALTH: Consolidated Standards of Reporting Trials of Electronic and Mobile Health Applications and Online Telehealth
RCT: randomized controlled trial
STI: sexually transmitted infection
