# Supplementary material for: Comparing the Efficacy of 2 WeChat Mini Programs in Reducing Nonmarital Heterosexual Contact by Male Factory Workers: Randomized Controlled Trial
Source: J Med Internet Res. 2024 Sep 9;26:e49362. doi: 10.2196/49362 (PMC11420611; doi:10.2196/49362)
Supplement: Multimedia Appendix 1 [file jmir_v26i1e49362_app1.docx]

Multimedia Appendix 1. Comparing baseline characteristics between participants who completed follow-up evaluation at T1 and those who were lost to follow up

|  | Intervention group | | | Control group | | |
| --- | --- | --- | --- | --- | --- | --- |
|  | Participants who completed T1 follow-up (n=117) | Participants who were lost to follow up at T1 (n=8) | *P* values | Participants who completed T1 follow-up (n=104) | Participants who were lost to follow up at T1 (n=18) | *P* values |
|  | n (%) | n (%) |  | n (%) | n (%) |  |
| **Sociodemographic characteristics** |  |  |  |  |  |  |
| Age group, years |  |  |  |  |  |  |
| 18-30 | 46 (39.3) | 1 (12.5) |  | 36 (34.6) | 9 (50.0) |  |
| 31-40 | 56 (47.9) | 6 (75.0) |  | 44 (42.3) | 6 (33.3) |  |
| 41-50 | 12 (10.3) | 1 (12.5) |  | 19 (18.3) | 1 (5.6) |  |
| >50 | 3 (2.6) | 0 (0.0) | .43 | 5 (4.8) | 2 (11.1) | .28 |
| Ethnicity |  |  |  |  |  |  |
| Han | 111 (94.9) | 7 (87.5) |  | 96 (92.3) | 17 (94.4) |  |
| Minority | 6 (5.1) | 1 (12.5) | .38 | 8 (7.7) | 1 (5.6) | .75 |
| Registered permanent residents of Shenzhen |  |  |  |  |  |  |
| No | 103 (88.0) | 8 (100.0) |  | 100 (96.2) | 15 (83.3) |  |
| Yes | 14 (12.0) | 0 (0.0) | .30 | 4 (3.8) | 3 (16.7) | .03 |
| Relationship status |  |  |  |  |  |  |
| Currently single | 38 (32.5) | 3 (37.5) |  | 29 (27.9) | 4 (22.2) |  |
| Having a stable girlfriend/married to a woman | 79 (67.5) | 5 (62.5) | .77 | 75 (72.1) | 14 (77.8) | 0.62 |
| Education level |  |  |  |  |  |  |
| Junior high or below | 18 (15.4) | 5 (62.5) |  | 23 (22.1) | 4 (22.2) |  |
| Senior high or equivalent | 29 (24.8) | 2 (25.0) |  | 26 (25.0) | 5 (27.8) |  |
| College and above | 70 (59.8) | 1 (12.5) | .002 | 55 (52.9) | 9 (50.0) | .97 |
| Living with either partner or spouse or children in Shenzhen |  |  |  |  |  |  |
| No | 51 (43.6) | 5 (62.5) |  | 47 (45.2) | 5 (27.8) |  |
| Yes | 66 (56.4) | 3 (37.5) | .30 | 57 (54.8) | 13 (72.2) | .17 |
| Monthly personal income, ¥ (US$) |  |  |  |  |  |  |
| <3000 (469.5) | 7 (6.0) | 2 (25.0) |  | 3 (2.9) | 0 (0.0) |  |
| 3000-4999 (469.5-782.3) | 31 (26.5) | 0 (0.0) |  | 29 (27.9) | 9 (50.0) |  |
| 5000-9999 (782.9-1564.8) | 68 (58.1) | 6 (75.0) |  | 68 (65.4) | 9 (50.0) |  |
| ≥10,000 (1564.9) | 11 (9.4) | 0 (0.0) | .07 | 4 (3.8) | 0 (0.0) | .24 |
| Status as frontline workers or management staff |  |  |  |  |  |  |
| Frontline workers | 77 (65.8) | 5 (62.5) |  | 72 (69.2) | 13 (72.2) |  |
| Management staff | 40 (34.2) | 3 (37.5) | .85 | 32 (30.8) | 5 (27.8) | .80 |
| **HIV or STI prevention service utilization in the past six months** |  |  |  |  |  |  |
| Use of HIV testing |  |  |  |  |  |  |
| No | 112 (95.7) | 7 (87.5) |  | 98 (94.2) | 18 (100.0) |  |
| Yes | 5 (4.3) | 1 (12.5) | .29 | 6 (5.8) | 0 (0.0) | .30 |
| Use of other HIV or STI prevention services (receiving free condoms, pamphlets, or attending workshops/seminars) |  |  |  |  |  |  |
| No | 87 (74.4) | 8 (100.0) |  | 86 (82.7) | 17 (94.4) |  |
| Yes | 30 (25.6) | 0 (0.0) | .10 | 18 (17.3) | 1 (5.6) | .20 |
| **Sexual behaviors in the past six months** |  |  |  |  |  |  |
| Sexual intercourse with non-regular female sex partners (NRP) |  |  |  |  |  |  |
| No | 73 (62.4) | 5 (62.5) |  | 65 (62.5) | 14 (77.8) |  |
| Yes | 44 (37.6) | 3 (37.5) | 1.00 | 39 (37.5) | 4 (22.2) | .21 |
| Sexual intercourse with female sex workers (FSW) |  |  |  |  |  |  |
| No | 72 (61.5) | 5 (62.5) |  | 70 (67.3) | 14 (77.8) |  |
| Yes | 45 (38.5) | 3 (37.5) | .96 | 34 (32.7) | 4 (22.2) | .38 |
| Condomless sex with NRP (among participants who had sexual intercourse with NRP at baseline) |  |  |  |  |  |  |
| No | 15 (34.1) | 2 (66.7) |  | 26 (41.0) | 1 (25.0) |  |
| Yes | 29 (65.9) | 1 (33.3) | .26 | 23 (59.0) | 3 (75.0) | .53 |
| Condomless sex with FSW (among participants who had sexual intercourse with FSW at baseline) |  |  |  |  |  |  |
| No | 13 (28.9) | 1 (33.3) |  | 8 (23.5) | 0 (0.0) |  |
| Yes | 32 (71.1) | 2 (66.7) | .87 | 26 (76.5) | 4 (100.0) | .28 |
