# Supplementary material for: Comparing the Efficacy of 2 WeChat Mini Programs in Reducing Nonmarital Heterosexual Contact by Male Factory Workers: Randomized Controlled Trial
Source: J Med Internet Res. 2024 Sep 9;26:e49362. doi: 10.2196/49362 (PMC11420611; doi:10.2196/49362)
Supplement: Multimedia Appendix 2 [file jmir_v26i1e49362_app2.docx]

Appendix 2. Comparing baseline characteristics between participants who completed follow-up evaluation at T2 and those who were lost to follow up

|  | Intervention group | | | Control group | | |
| --- | --- | --- | --- | --- | --- | --- |
|  | Participants who completed T2 follow-up (n=116) | Participants who were lost to follow up at T2 (n=9) | *P* values | Participants who completed T2 follow-up (n=104) | Participants who were lost to follow up at T2 (n=18) | *P* values |
|  | n (%) | n (%) |  | n (%) | n (%) |  |
| **Sociodemographic characteristics** |  |  |  |  |  |  |
| Age group, years |  |  |  |  |  |  |
| 18-30 | 44 (37.9) | 3 (33.3) |  | 37 (35.6) | 8 (44.4) |  |
| 31-40 | 57 (49.1) | 5 (55.6) |  | 43 (41.3) | 7 (38.9) |  |
| 41-50 | 12 (10.3) | 1 (11.1) |  | 19 (18.3) | 1 (5.6) |  |
| >50 | 3 (2.6) | 0 (0.0) | .95 | 5 (4.8) | 2 (11.1) | .40 |
| Ethnicity |  |  |  |  |  |  |
| Han | 111 (95.7) | 7 (77.8) |  | 96 (92.3) | 17 (94.4) |  |
| Minority | 5 (4.3) | 2 (22.2) | .08 | 8 (7.7) | 1 (5.6) | .75 |
| Registered permanent residents of Shenzhen |  |  |  |  |  |  |
| No | 102 (87.9) | 9 (100.0) |  | 100 (96.2) | 15 (83.3) |  |
| Yes | 14 (12.1) | 0 (0.0) | .27 | 4 (3.8) | 3 (16.7) | .03 |
| Relationship status |  |  |  |  |  |  |
| Currently single | 37 (31.9) | 4 (44.4) |  | 28 (26.9) | 5 (27.8) |  |
| Having a stable girlfriend/married to a woman | 79 (68.1) | 5 (55.6) | .44 | 76 (73.1) | 13 (72.2) | .94 |
| Education level |  |  |  |  |  |  |
| Junior high or below | 18 (15.5) | 5 (55.6) |  | 23 (22.1) | 4 (22.2) |  |
| Senior high or equivalent | 30 (25.9) | 1 (11.1) |  | 25 (24.0) | 6 (33.3) |  |
| College and above | 68 (58.6) | 3 (33.3) | .01 | 56 (53.8) | 8 (44.4) | .68 |
| Living with either partner or spouse or children in Shenzhen |  |  |  |  |  |  |
| No | 49 (42.2) | 7 (77.8) |  | 46 (44.2) | 6 (33.3) |  |
| Yes | 67 (57.8) | 2 (22.2) | .08 | 58 (55.8) | 12 (66.7) | .39 |
| Monthly personal income, ¥ (US$) |  |  |  |  |  |  |
| <3000 (469.5) | 7 (6.0) | 2 (22.2) |  | 3 (2.9) | 0 (0.0) |  |
| 3000-4999 (469.5-782.3) | 30 (25.9) | 1 (11.1) |  | 28 (26.9) | 10 (55.6) |  |
| 5000-9999 (782.9-1564.8) | 68 (58.6) | 6 (66.7) |  | 69 (66.3) | 8 (44.4) |  |
| ≥10,000 (1564.9) | 11 (9.5) | 0 (0.0) | .19 | 4 (3.8) | 0 (0.0) | .09 |
| Status as frontline workers or management staff |  |  |  |  |  |  |
| Frontline workers | 77 (66.4) | 5 (55.6) |  | 73 (70.2) | 12 (66.7) |  |
| Management staff | 39 (33.6) | 4 (44.4) | .51 | 31 (29.8) | 6 (33.3) | .76 |
| **HIV or STI prevention service utilization in the past six months** |  |  |  |  |  |  |
| Use of HIV testing |  |  |  |  |  |  |
| No | 112 (96.6) | 8 (88.9) |  | 99 (95.2) | 18 (100.0) |  |
| Yes | 4 (3.4) | 1 (11.1) | .26 | 5 (4.8) | 0 (0.0) | .34 |
| Use of other HIV or STI prevention services (receiving free condoms, pamphlets, or attending workshops/seminars) |  |  |  |  |  |  |
| No | 87 (75.0) | 8 (88.9) |  | 86 (82.7) | 17 (94.4) |  |
| Yes | 29 (25.0) | 1 (11.1) | .35 | 18 (17.3) | 1 (5.6) | .20 |
| **Sexual behaviors in the past six months** |  |  |  |  |  |  |
| Sexual intercourse with non-regular female sex partners (NRP) |  |  |  |  |  |  |
| No | 71 (61.2) | 7 (77.8) |  | 66 (63.5) | 13 (72.2) |  |
| Yes | 45 (38.8) | 2 (22.2) | .32 | 38 (36.5) | 5 (27.8) | .47 |
| Sexual intercourse with female sex workers (FSW) |  |  |  |  |  |  |
| No | 70 (60.3) | 7 (77.8) |  | 70 (67.3) | 14 (77.8) |  |
| Yes | 46 (39.7) | 2 (22.2) | .30 | 34 (32.7) | 4 (22.2) | .38 |
| Condomless sex with NRP (among participants who had sexual intercourse with NRP at baseline) |  |  |  |  |  |  |
| No | 16 (35.6) | 1 (50.0) |  | 15 (39.5) | 2 (40.0) |  |
| Yes | 29 (64.4) | 1 (50.0) | .68 | 23 (60.5) | 3 (60.0) | .98 |
| Condomless sex with FSW (among participants who had sexual intercourse with FSW at baseline) |  |  |  |  |  |  |
| No | 14 (30.4) | 0 (0.0) |  | 8 (23.5) | 0 (0.0) |  |
| Yes | 32 (69.6) | 2 (100.0) | .35 | 26 (76.5) | 4 (100.0) | .28 |
